# Impact of Pyrolysis Temperature on the Properties of Eucalyptus Wood-Derived Biochar

**DOI:** 10.3390/ma13245841

**Published:** 2020-12-21

**Authors:** Bruno Caio Chaves Fernandes, Kassio Ferreira Mendes, Ananias Francisco Dias Júnior, Vinícius Patrício da Silva Caldeira, Taliane Maria da Silva Teófilo, Tatiane Severo Silva, Vander Mendonça, Matheus de Freitas Souza, Daniel Valadão Silva

**Affiliations:** 1 Departamento de Ciências Agronômicas e Florestais, Centro de Ciências Vegetais, Universidade Federal Rural do Semi-Árido, Av. Francisco Mota, 572, Costa e Silva, 59625-900 Mossoró, Brazil; Talianeteofilo23@gmail.com (T.M.d.S.T.); Tatiane.severosilva@gmail.com (T.S.S.); vander@ufersa.edu.br (V.M.); Matheus_mafs10@hotmail.com (M.d.F.S.); Daniel.valadao@ufersa.edu.br (D.V.S.); 2 Departamento de Ciências Agronômicas, Universidade Federal de Viçosa (UFV), 36570-900 Viçosa, Brazil; kassio_mendes_06@hotmail.com; 3 Departamento de Ciências Florestais e da Madeira, Universidade Federal do Espírito Santo (UFES), 29550-000 Jerônimo Monteiro, Brazil; ananiasjr@usp.br; 4 Departamento de Química, Universidade Estadual do Rio Grande do Norte (UERN), 59600-195 Mossoró, Brazil; Vinicius_psc@yahoo.com.br

**Keywords:** adsorbent materials, characterization, chemical composition, eucalyptus waste and surface functional group

## Abstract

Pyrolysis conditions directly influence biochar properties and, consequently, influence the potential use of biochar. In this study, we evaluated the effects of different pyrolysis temperatures (450, 550, 650, 750, 850, and 950 °C) on the hydrogen potential, electrical conductivity, ash content, yield, volatile matter content, elemental analysis, Fourier-transform infrared spectroscopy results, X-ray diffraction results, scanning electron microscopy results, specific surface area, and micropore volume of eucalyptus wood-derived biochar. The degree of linear association between pyrolysis temperatures and biochar properties was examined using the Pearson correlation coefficient. The results showed a positive correlation of the pyrolysis temperature with the hydrogen potential value, electrical conductivity, and elemental carbon. There was a negative correlation of the pyrolysis temperature with the yield, volatile matter content, elemental oxygen, elemental hydrogen, surface area, aromaticity, hydrophilicity, and polarity indexes. The Fourier-transform infrared spectroscopy data indicated an increase in aromaticity and a decrease in the polarity of high-temperature biochar. The increased pyrolysis temperature caused the loss of cellulose and crystalline mineral components, as indicated by X-ray diffraction analysis and scanning electron microscopy images. These results indicated that changing the pyrolysis temperature enables the production of biochar from the same raw material with a wide range of physicochemical properties, which allows its use in various types of agricultural and environmental activities.

## 1. Introduction

Biochar is a carbon-rich porous material produced by biomass pyrolysis under an environment with limited oxygen or anoxic conditions [1,2]. Its use is a potential alternative that can promote agricultural yield benefits, carbon sequestration, waste management, clean energy production, and reclamation of degraded areas [3,4,5].

A wide variety of biomass feedstocks can be used to produce biochar. Large amounts of agricultural waste are generated worldwide and are not always properly discarded or recycled. Wood production in Brazil annually generates approximately 50.8 million m^3^ of lignocellulosic waste [6]; eucalyptus is the most cultivated forest species with 7.83 million hectares of planted trees [7] and contributes largely to the creation of this lignocellulosic waste. Waste conversion into biochar by pyrolysis can reduce lignocellulosic waste volume and contribute to power generation, improvements in crop nutrient efficiency, elimination of pathogens, and generation of products with high agronomic and environmental value [8].

Pyrolysis can be carried out in different temperature ranges, heating rates, and residence times of biomass. These conditions promote wide variation in yield [9] and the properties of the produced biochar [10]. Even produced from the same material, the pyrolysis conditions can produce biochar with different chemical-physical characteristics and textures [11,12] and can thus change its efficiency or purpose of use.

Several studies have evaluated the relationships between various biochars and their pyrolysis conditions [13,14,15]. Huff et al. (2014) [16] reported an increase in aromaticity of biochars from pine wood, peanut shell, and bamboo biomass at higher temperatures, and they reported on the possibility of using these biochars for the potential removal of organic molecules. Jones et al. (2011) [17] studied the effect of biochar from eucalyptus wood (*Eucalyptus marginatus*) on simazine leaching and degradation and noted that a high sorption of this product reduced its mobility; however, herbicide persistence was increased with the reduced availability of microorganisms in soil.

The use of biochar produced from eucalyptus residues, which is an abundant material and is easy to acquire in several countries [18], can improve the destination of residues from the forestry industry. However, as reported, the temperature of pyrolysis influences the properties of biochar, which can influence the effectiveness of its use. Thus, the objective of this research was to determine the effect of pyrolysis temperature on the physicochemical characteristics of the eucalyptus waste biochar in order to inform biochar producers and stakeholders about the most suitable characteristics for agronomic and environmental applications.

## 2. Material and Methods

### 2.1. Biochar Production

Eucalyptus residue was acquired from a clone wood resulting from the crossing between *Eucalyptus urophylla* and *Eucalyptus grandi*. The material was crushed into small pieces using a TECNAL knife mill (Willey TE 340, Piracicaba, Brazil). Then, the biomass powder was passed through a 2 mm sieve and dried in an oven with air circulation at 103 ± 2 °C. Biomass pyrolysis was performed in a metal cylinder inside a muffle furnace (Q318M, Quimis, Diadema, São Paulo, Brazil). Carbonization was performed at final temperatures of 450, 550, 650, 750, 850, and 950 °C. The amount of residue used for each repetition was 200 ± 10 g; the temperature was increased by 1.7 °C min^−1^ and maintained at the final temperature for 1 h under a limited air supply [19]. After cooling, the biochar samples were ground, sieved (<0.9 mm) and stored in a desiccator. The biochar yield was calculated by its mass difference in relation to the dry wood. 

### 2.2. Biochar Characterization

#### 2.2.1. Elemental and Energy Dispersion X-ray Fluorescence Spectrometry Analyses

An elemental analyzer was used to determine the amount of carbon (C), oxygen (O), hydrogen (H), and nitrogen (N) (Vario MACRO Cube, Elementar, Cheadle Hulme, United Kingdom). One hundred milligrams of each dried biochar sample was weighed in a tin capsule; next, the capsule was closed and placed in an apparatus, which was operated by subjecting the samples to combustion under a pure oxygen atmosphere (99.999%). Gases formed by this combustion were measured with a thermal conductivity detector. The O content was estimated by mass balance: O = 100 − (C + H + N + mineral).

The inorganic mineral content was determined by energy-dispersive X-ray fluorescence spectrometry (EDXRF). One gram of the biochar sample was weighed and ground; next, the biochar sample was packed in a 20 mm-diameter polyethylene beaker and covered with a 6 µm thick polypropylene film. The samples were irradiated in triplicate for 300 s under vacuum using an EDXRF instrument (EDXRF-720, Shimadzu, Kyoto, Japan). The samples were irradiated using an X-ray tube operated at 15 kV (sodium a scandium) and 50 kV (titanium a uranium). The current was adjusted automatically (maximum: 1 mA) using a 10 mm collimator. Detection was performed with a liquid nitrogen-cooled Si (Li) detector (Shimadzu, Kyoto, Japan).

#### 2.2.2. Ash and Volatile Matter (VM) Contents 

Ash and VM contents were evaluated by using the American Society for Testing and Materials (ASTM) methods. Each sample was analyzed in triplicate. For ash analysis, porcelain crucibles were preheated in an oven at 650 °C for 10 min; next, the crucibles were cooled in a desiccator at room temperature and preweighed. The ash content was determined by measuring the mass loss after the combustion of 1 g of biochar sample in each porcelain crucible at 105 °C for 1 h and 750 °C for 6 h [20].

The VM content was determined by heating the dried sample at 900 °C in a muffle furnace (SP-1200DM/B, Splabor, Presidente Prudente, São Paulo, Brazil). Approximately 1 g of the dried biochar sample was weighed and placed in a preweighed and preheated porcelain crucible. The crucible was placed in the preheated muffle door at 900 °C for 3 min and then placed inside a muffle furnace for 7 min. After the oven was cooled, the samples were removed and placed in a desiccator at room temperature and then weighed. The VM content was determined by the mass difference in the crucible before being placed and after being removed from the muffle furnace [21].

#### 2.2.3. pH Value and Electrical Conductivity (EC)

For pH measurements, 2 g of each biochar sample was weighed into a 25 mL beaker; next, 10 mL of a 0.01 M CaCl_2_ was added to a beaker and stirred with a glass rod; and then, the mixture was allowed to sit for 1 h [22]. The pH measurement was performed with a digital pH meter (Mpa-210, Tecnopon, Piracicaba, São Paulo, Brazil).

The EC was determined by the BGK method [23]. A solution of 2 g of dry biochar and 10 mL of deionized water was prepared and stirred for 1 h. Then, the measurements were performed on a digital conductivity meter (MA 521, Marconi, Piracicaba, São Paulo, Brazil). The pH and EC analyses were performed in triplicate.

#### 2.2.4. Specific Surface Area (SSA) and Morphology

Scanning electron microscopy (SEM) was used to evaluate the changes in the physical morphology of the biochar surface caused by variations in pyrolysis temperature. The biochar particles were placed in a metal sample holder using a carbon conductive tape (PELCO Tabs™, Ted Pella, Inc., Redding, CA, USA) and sputtered (Q150R ES, Quorum Technologies Ltd., Laughton, East Sussex, UK) with a 9 nm thick gold layer. This procedure was used to improve material conductivity, generating a higher-quality image. Images were captured with a secondary electron detector (SE) in SEM (VEGA 3 LMU, Tescan, Czech Republic), operating with an electron beam of 20 kV.

The textural properties of the biochar surface were characterized with the Brunauer–Emmett–Teller (BET) method by using results from the physical adsorption of N_2_ at 77 K with the aid of a surface area analyzer (ASAP-2020, Micromeritics, Norcross, GA, USA) at a relative pressure range (P/P_0_) of 0.01–0.99. At each pressure, the physically adsorbed N_2_ produced a change in the output composition recorded by a thermal conductivity detector connected to a potentiometric recorder. By heating the sample, there was a loss in contact with the sample liquid N_2_, and the N_2_ was desorbed. The peak area is proportional to the mass of the desorbed N_2_. From the N_2_ volume obtained in the assay and using the BET equation, the SSA and total pore volume (TPV) were determined.

#### 2.2.5. Chemical Analysis

The spatial arrangements and presence of minerals in the samples were identified using the X-ray diffraction (XRD) technique; the bands were scanned in a 2θ range from 10° to 80° at low and medium angles (XRD 6000, Shimadzu, Quioto, Japan) with a radiation source of CuKα (λ  = 0.15406 nm) and a nickel filter operating at 30 kV and a rate of 30 mA at 0.6° min^−1^.

The surface functional groups were analyzed by Fourier-transform infrared spectroscopy (FT-IR) in a wavelength range of 400–4000 cm^−1^ (by 64 scans with a 4 cm^−1^ resolution). Before FT-IR analysis, the samples were oven-dried at 200 °C, and then 0.5 mg of the sample was mixed with 300 mg of KBr and ground. Thereafter, each sample was pressed in a hydraulic press (Shimadzu, SSP-10A, Tokyo, Japan) at an 80 kN force to form a tablet that was analyzed on a spectrometer (IRPrestige-21, Shimadzu, Tokyo, Japan).

Due to the need for a relative scale of the absorption intensity, a background spectrum was measured, which aimed to compare the sample-free beam measurement to the sample-beam measurement to determine the percent transmittance. All spectral information was strictly due to the sample. As the background spectrum is a feature of the instrument, only one measurement of this spectrum was performed.

### 2.3. Data Analysis

The degree of linear association between two measured variables was analyzed using the Pearson correlation coefficient. The relationships between pyrolysis conditions (independent variables) and biochar-derived properties (dependent variables) were evaluated. A significance rate was considered (*p* ≤ 0.05). R values were calculated using Statistica^®^ software version 7.0 (StatSoft, Boston, MA, USA).

## 3. Results and Discussion

### 3.1. Effects of Pyrolysis Temperature on Yield, VM Content, Ash Content, pH Value, EC, and Inorganic Minerals from Biochar Produced

A significantly negative correlation occurred between the pyrolysis temperature and the biochar yield (R = −0.90), indicating a decrease in yield due to the increased pyrolysis temperature (Table 1). The yield declined sharply, as the temperature increased from 450 to 650 °C (Table 1). The VM content showed a similar behavior to yield, and there was an exponential reduction in VM content from 450 to 650 °C and a significant correlation of −0.90 (Table 1). The main transformation observed over this temperature range was the thermal decomposition of lignocellulosic materials and the release of water vapor and VM compounds such as CO, CO_2_, H_2_, and CH_4_, resulting in a high yield loss at lower temperatures [24,25]. Between 750 and 850 °C, the decline in biochar yield was proportionally lower and constant, indicating the formation of the most stable carbonaceous compound in terms of material loss. Other studies have shown a similar correlation for other materials reacted at different pyrolysis temperatures; that is, other materials show a rapid initial decline in yield at lower temperatures, followed by a smaller and constant reduction at higher temperatures [26,27,28,29].

Unlike yield and VM content, ash content, pH values, and EC increased due to the temperature increase, with significant positive correlations of 0.89, 0.83, and 0.85, respectively (Table 1). Higher ash contents observed at higher temperatures were caused by the stability of minerals in the pyrolyzed product, such as K, Ca, and P, following a progressive loss in VM from the devolatilization process [30]. The positive correlation of these minerals explained this observation with the temperature increase (Table 2). Another observed effect associated with higher ash content is the increase in pH value and EC. Lehmann and Joseph (2015) [31] developed a meta-analysis and reported a close relationship between pyrolysis temperature and pH value. These authors observed that the average pH value of pyrolyzed biochar was 5 at temperatures of <400 °C, while the pH value of pyrolyzed biochar was 9 at temperatures of >600 °C. The ability of biochar to neutralize the acidity has been acknowledged in several studies, showing biochar is a potential agent for correcting pH values in acidic soils, reducing the toxicity of aluminum and increasing the availability of nutrients [32,33].

In addition to the pH value, increased cation concentrations due to the pyrolysis temperature increase favored higher ECs of the material. Biochars with the highest concentration of salts can be a problem in areas with the limited ability to leach salts. The application of biochar with high EC values can reduce seed germination and crop yields with higher sensitivity to salt in a soil solution [34]. However, most studies showed a positive correlation between biochar use and lower crop toxicity due to water and saline stress [35,36,37,38]. The increase in biochar EC due to a higher pyrolysis temperature was mainly caused by a higher K concentration observed in this work (Table 2). The K supply via the biochar application can improve osmotic control and potassium/sodium ratios, which increases the crop tolerance to water and saline stress [39].

### 3.2. Elemental Analysis

The pyrolysis temperature changed the elemental composition of the eucalyptus wood waste-derived biochar (Table 3). Increasing the temperature from 450 to 950 °C increased the C content from 74.96% to 86.93%, respectively, with a significant positive correlation (R = 0.84) (Table 2). The H and O contents were negatively correlated with the increase in pyrolysis temperature (R = 0.72 and 0.84, respectively), with a decrease of 3.76% to 0.9% in H content and 19.41% to 10.75% in O content. The gradual increase in C content of the biochar due to the increase in temperature was caused by a higher release rate of hydrogen and oxygen in the form of water vapor (dehydration) and the loss of functional groups associated with these atoms (carboxyl groups and hydroxyls). During the carbonization process, the relative loss in hydrogen and oxygen is higher than the relative loss in carbon [30]; this behavior was also observed for the eucalyptus-derived biochar, changing the atomic ratios of biochars.

C, elemental carbon; H, elemental hydrogen; O, elemental oxygen; N, elemental nitrogen; H/C, aromaticity index; (O/C), hydrophilicity index; (O + N)/C, polarity index. * indicates significant correlations (*p* ≤ 0.05). N = 3; data are presented as mean ± stand deviation.

The elemental oxygen (O) (R = 0.84) and hydrogen (H) (R = 0.84) positively correlated with the pyrolysis temperature. The O/C ratio significantly negatively correlated with the increase in temperature (R = −0.85) (Table 3). The correlation with the H/C and (O + N)/C ratios was not significant, although the correlation showed high values (R = −0.8 and −0.81, respectively) (Table 2). The reduction in atomic ratios due to the increased temperature pyrolysis indicated that the material formed had higher aromaticity and recalcitrance values but a smaller polarity [4,26,40]. These properties ensure that biochar has a greater resistance to degradation when applied to soils [12,41,42]. For the biochar derived from eucalyptus produced at 850 and 950 °C, the increased stability of these carbonaceous materials provided carbon sequestration in soil since they were hardly changed in their elemental structure. Despite the lower H/C and O/C ratios of biochars produced at pyrolysis temperatures of 850 and 950 °C, all the other biochars produced at the lowest temperatures in this research were within the parameters required by the European Biochar Certificate [43]: C% > 50, H/C ratio < 0.7, and O/C ratio < 0.4.

The pyrolysis temperature did not change the N content, showing a nonsignificant correlation (R = 0.08) (Table 3). Generally, the N content depends on the type of raw material used for biochar production and does not rely on the pyrolysis conditions [4,44]. Moreover, in woody matrices, N-containing structures are more resistant to degradation with increasing temperature. According to Enders et al. 2012 [18], N is highly retained in raw material wood-derived biochars, which is probably due to the formation of heterocyclic N such as pyridines and pyrroles.

**Figure 1:**
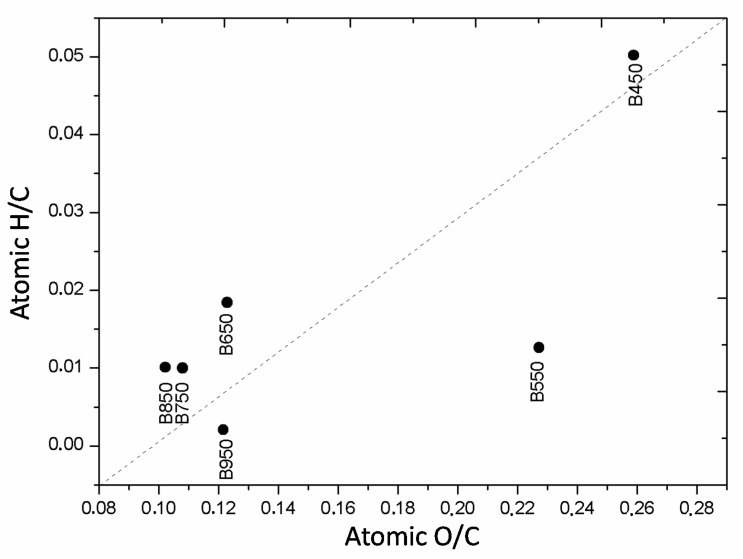
Van Krevelen diagram showing the correlations between the H/C and O/C ratios of pyrolyzed eucalyptus wood-derived biochar at different temperatures.

The van Krevelen diagram allows us to identify changes in the H/C and O/C ratios in eucalyptus-derived biochar (Figure 1). An increase in the pyrolysis temperature from 450 to 550 °C promoted an intense reduction in aliphatic groups (H/C). At temperatures above 550 °C, the aliphatic degree of biochar changed little. The main change from 550 °C was the reduction in the O/C ratio, providing biochar with a greater similarity for the H/C and O/C ratios. Pyrolysis at 950 °C promoted another decrease in the H/C ratio. This behavior observed for the higher pyrolysis temperatures suggested the beginning of the formation process of a carbonaceous material with a high amount of metamorphism called coal anthracite [45].

### 3.3. SSA, TPV, Surface Morphology, Crystalline Phase, and Functional Groups

The pyrolysis temperature showed a significant negative correlation with the biochar SSA (R = −0.89) (Table 3). As the pyrolysis temperature increased from 650 to 950 °C, the SSA decreased from 410.48 to 224.43 m^2^ g^−1^. This reduction in the SSA due to material burning at higher temperatures is not common. Most studies show that a higher pyrolysis temperature provides a higher SSA [4,46]. For eucalyptus-derived biochar, the reduction in the SSA can be attributed to the higher ash content observed at temperatures above 650 °C (Table 1), which can decrease the microporous formation structure [14]. In addition, micropores can be destroyed by the polymerization reaction during the heating process [47], resulting in a lower SSA found in biochar produced at higher pyrolysis temperatures.

**Figure 2:**
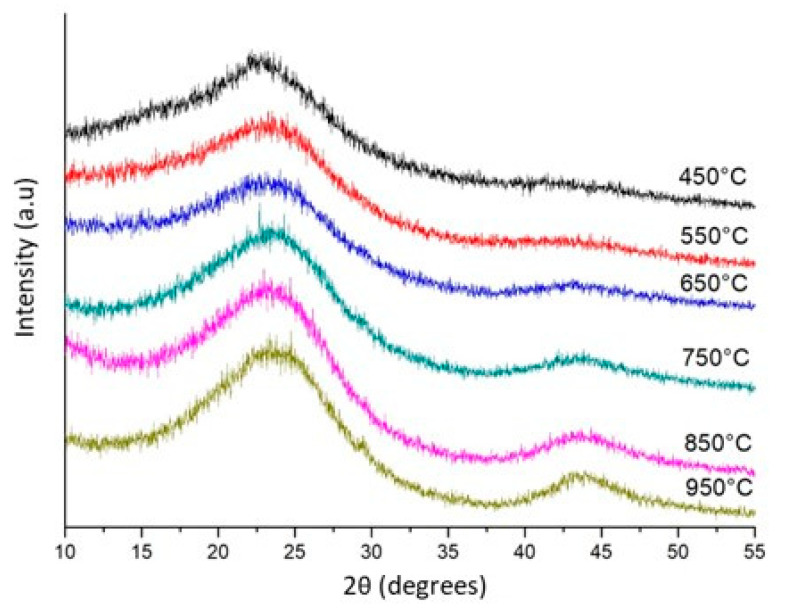
Diffracted beam intensity as a function of the Bragg angle (2θ in degrees) of eucalyptus wood-derived biochar at different pyrolysis temperatures.

The increased pyrolysis temperature showed no significant correlation with the TPV (R = −0.34). The TPV increased from 0.05 to 0.07 mm^3^ g^−1^ with the temperature increased from 650 to 850 °C and decreased to 0.02 mm^3^ g^−1^ at 950 °C. Increasing the temperature to 950 °C may have destroyed or blocked the micropores. Moreover, the XRD analysis results showed that the microcrystalline graphite structure was more present in high-temperature biochars (Figure 2). The process of converting amorphous carbon into graphite microcrystalline structures can cause decreases in the TPV and SSA, as reported by Chen et al. 2012 [48], when evaluating the effect of pyrolysis temperature on cotton stalks. At temperatures of 450 and 550 °C, the device could not determine the SSA and TPV. The amount of uncarbonized biomass at these temperatures may have interfered with the measurements, and this prevents the gas adsorption measurements (N_2_-BET), which allow for the estimation of the surface area and pore volume [49,50]. 

**Figure 3:**
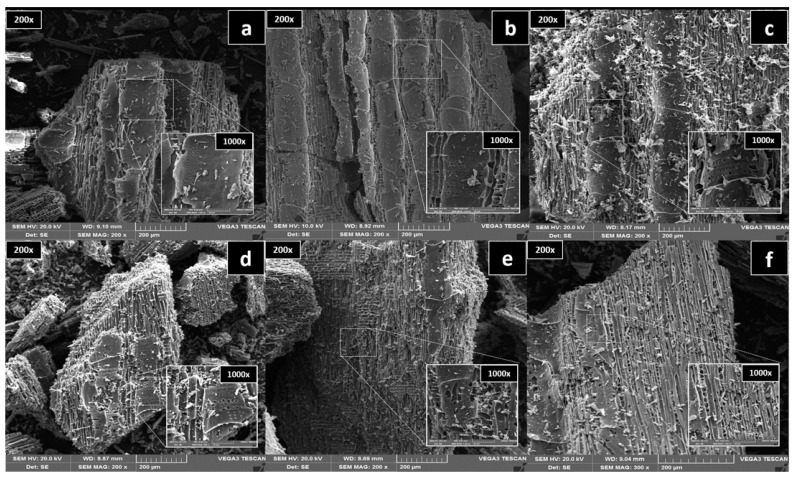
Scanning electron microscopy (SEM) images of eucalyptus wood-derived biochar produced at 200× and 1000× magnifications at different pyrolysis temperatures: (**a**) 450 °C; (**b**) 550 °C; (**c**) 650 °C; (**d**) 750 °C; (**e**) 850 °C; and (**f**) 950 °C.

The SEM images at 200× and 1000× magnifications showed that increasing the pyrolysis temperature promoted the loss of the outer biochar layer (Figure 3). The removal of the outer biochar layer exposed pores located in the carbonaceous skeleton of the biological capillary structure of the raw material. A greater number of porous structures were observed with increasing pyrolysis temperature (Figure 3). However, the accumulation of particles on the biochar surface, mainly ash, was also increased due to the higher temperature (Figure 3). The presence of this greater amount of ash on the surface of biochars produced at higher temperatures justifies the evidence that these particles could clog the micropores, reducing both the SSA and TPV measured with the BET analysis.

The crystalline phase shape and biochar purity were determined using XRD analysis. The spectrum showed no sharp peaks representing the presence of crystalline mineral structures (Figure 2). The XRD peak intensities increased with increasing pyrolysis temperature. The 2θ angle peak at approximately 22.7° was attributed to the crystallographic planes hkl (101) of the crystalline regions of cellulose [51,52]. Pyrolysis temperatures above 650 °C resulted in a wider peak at a 2θ value of approximately 23.5°, indicating that some partial crystalline structure of the cellulose was lost [26]. A new 2θ peak of approximately 43.7° became clearer in biochars at pyrolysis temperatures of 750 to 950 °C. This new observed peak indicated the development of graphite-like atomic order in the increasingly charred material [26]. This peak resulted in the successive ordering of carbon in aromatic structures [53], indicating the highest crystallization of the carbonaceous material [54]. These findings point to the formation of more stable (less reactive) graphitic carbon at higher pyrolysis temperatures.

**Figure 4:**
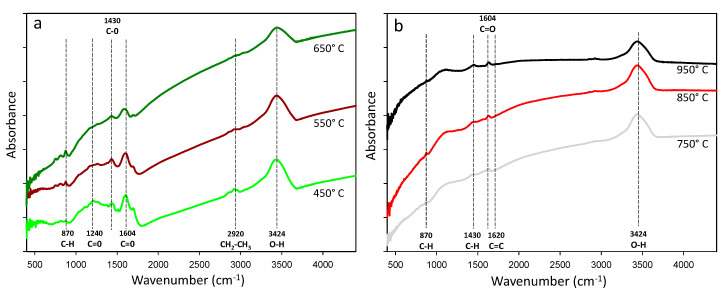
Fourier-transform infrared spectra (FT-IR) of eucalyptus wood-derived biochar samples at different pyrolysis temperatures (**a**) 450, 550 and 650 °C and (**b**) 750, 850 and 950 °C.

The functional groups observed in the eucalyptus wood residue-derived biochar by FT-IR analysis are shown in Table 4. Aromatic, aliphatic, and phenolic groups were seen as the predominant peaks. A high intensity peak at 3.424 cm^−1^, corresponding to the stretching vibrations of the –OH groups, became weaker with increasing temperature (Table 3 and Figure 4). There was a reduction in peaks from 1000 to 1700 cm^−1^ with increasing pyrolysis temperature (Figure 4). At lower temperatures (450, 550, and 650 °C), peaks at 1.604 cm^−1^ (C = O) [55] and 1.240 cm^−1^ (C–O acetyls) were observed, suggesting the presence of woody and cellulosic constituents and the incomplete degradation of the original biomass [13].

The peak at 2.920 cm^−1^ (C–H, CH_2_, or CH_3_) [50] for aliphatic components was observed only at lower temperatures (450 and 550 °C) (Figure 3). With increasing pyrolysis temperature to above 550 °C, the aliphatic C-H stretching observed at peak of 2.920 cm^−1^ was eliminated, suggesting the degradation of hemicellulose and cellulose [40]. The peak representing the aromatic carbon at 1.638 cm^−1^ (C=C) [51,56] narrowed at the temperature of 750 °C (Table 4 and Figure 4). The higher peak intensity for the C=C group confirmed the increased aromaticity of the eucalyptus-derived biochar when produced at temperatures above 750 °C. The peak intensity at 1.430 cm^−1^ for C–H decreased dramatically with increasing temperature, reaching close to zero intensities when the material was pyrolyzed at 950 °C.

Increasing the pyrolysis temperature reduced the intensity of some biochar functional groups. This loss of functional groups, especially the hydroxyl and carboxyl groups, was caused by the degradation and transformation of hemicellulose (200–260 °C), cellulose (315–400 °C), and lignin (> 400 °C) in homogeneous and highly aromatic carbon structures [59]. The FT-IR spectra confirmed the transformation of the eucalyptus-derived material into more recalcitrant compounds when high pyrolysis temperatures were used during biochar synthesis. However, the absence of functional groups (carboxylic, phenolic, and alcohol) can make it impossible to use biochar for some specific purposes. The application of highly aromatic biochar without functional groups to reduce nutrient leaching [60] or remove soil contaminants [61] can be inefficient due to the absence of surface charges that are capable of interacting with nutrients or contaminants.

## 4. Conclusions

Some physicochemical properties of eucalyptus wood-derived biochar are affected by pyrolysis temperature. The results showed that the pyrolysis temperature was positively correlated with pH value, EC, ash content, and elemental carbon and negatively correlated with yield, VM, elemental oxygen, elemental hydrogen, surface area, and H/C, O/C, and (O + N)/C ratios. An increase in temperature promoted a loss in biochar surface layers, reduced surface functional groups and formed a more stable carbon. Overall, the research indicated that the pyrolysis temperature makes it possible to produce biochar from the same raw material with a wide range of physicochemical properties, allowing its use in various agricultural and environmental activities, such as soil conditioning, carbon sequestration, or the immobilization of contaminants.

## Figures and Tables

**Table 1:** Yield, ash, volatile matter (VM) contents, pH, and electrical conductivity (EC) of pyrolyzed eucalyptus wood-derived biochar at different temperatures.

Temperature (°C)	Yield (%)	Ash (%)	(VM) (%)	pH (CaCl_2_)	EC µS/cm
450	42.76 ± 0.54	0.60 ± 0.001	30.32 ± 1.09	5.3 ± 0.070	123.3 ± 1.414
550	38.00 ± 0.44	0.74 ± 0.012	16.49 ± 0.20	6.7 ± 0.142	97.4 ± 2.121
650	34.69 ± 0.79	0.63 ± 0.009	11.92 ± 0.50	9.7 ± 0.260	134.3 ± 1.697
750	34.27 ± 0.44	0.84 ± 0.002	5.52 ± 0.67	9.8 ± 0.035	164.6 ± 1.272
850	32.44 ± 0.37	0.82 ± 0.012	3.43 ± 0.65	9.1 ± 0.106	161.6 ± 1.187
950	33.47 ± 0.61	1.06 ± 0.031	3.63 ± 0.74	9.2 ± 0.084	273.1 ± 1.626
Correlation	−0.90 *	0.89 *	−0.90 *	0.83 *	0.85 *

* indicates significant correlations (*p* ≤ 0.05). N = 3; data are presented as mean ± stand deviation.

**Table 2:** Eucalyptus wood-derived biochar inorganic minerals produced at different pyrolysis temperatures.

Temperature (°C)	K (%)	Ca (%)	P (%)	Fe (%)	S (%)	Mn (%)	Zn (%)	Cr (%)	Si (%)
450	0.182 ± 0.001	0.085 ± 0.002	0.016 ± 0.002	0.001 ± 0.0003	0.014 ± 0.001	0.002 ± 0.0002			
550	0.140 ± 0.007	0.104 ± 0.001	0.016 ± 0.024	0.003 ± 0.0003	0.013 ± 0.001	0.003 ± 0.0001			0.045 ± 0.0007
650	0.164 ± 0.001	0.115 ± 0.004	0.023 ± 0.001	0.006 ± 0.0002	0.016 ± 0.0007	0.003 ± 0.0001	0.0002 ± 0.00006	0.002 ± 0.0001	
750	0.210 ± 0.003	0.130 ± 0.002	0.017 ± 0.001	0.006 ± 0.0007	0.010 ± 0.0007	0.003 ± 0.0002			
850	0.210 ± 0.004	0.104 ± 0.002	0.025 ± 0.0003	0.004 ± 0.0001	0.016 ± 0.0007	0.002 ± 0.0002			
950	0.286 ± 0.011	0.176 ± 0.004	0.031 ± 0.002	0.004 ± 0.0001	0.013 ± 0.0014	0.003 ± 0.0002			
Correlation	0.82 *	0.82 *	0.84 *	0.51	−0.05	0.21			

* indicates significant correlations (*p* ≤ 0.05). N = 3; data are presented as mean ± stand deviation.

**Table 3:** Elemental analysis, specific surface area (SSA), and total pore volume (TPV) of pyrolyzed eucalyptus wood biochar at different temperatures.

Temperature (°C)	Elemental Analysis							SSA and TPV	
	C (%)	H (%)	O (%)	N (%)	H/C	O/C	(O + N)/C	SSA (m^2^ g^−1^)	TPV (mm^3^ g^−1^)
450	74.96 ± 0.07	3.76 ± 0.38	19.41 ± 0.69	1.25 ± 0.21	0.050 ± 0.005	0.25 ± 0.009	0.27 ± 0.006		
550	79.25 ± 0.24	1.00 ± 0.14	18.01 ± 0.78	1.49 ± 0.68	0.012 ± 0.001	0.22 ± 0.01	0.25 ± 0.001		
650	87.06 ± 0.68	1.60 ± 0.41	10.70 ± 0.27	0.00	0.018 ± 0.004	0.12 ± 0.004	0.12 ± 0.004	410.48 ± 0.82	0.05 ± 0.002
750	87.74 ± 0.91	0.87 ± 0.10	9.47 ± 0.81	1.06 ± 0.007	0.019 ± 0.001	0.11 ± 0.01	0.12 ± 0.01	402.51 ± 0.91	0.07 ± 0.003
850	88.01 ± 1.03	0.89 ± 0.24	8.99 ± 0.25	1.27 ± 0.51	0.010 ± 0.002	0.10 ± 0.004	0.14 ± 0.01	362.90 ± 1.41	0.08 ± 0.003
950	86.90 ± 0.13	0.90 ± 0.04	10.57 ± 0.11	1.34 ± 0.183	0.002 ± 0.0005	0.12 ± 0.001	0.17 ± 0.01	224.43 ± 2.90	0.02 ± 0.002
Correlation	0.84 *	0.72	0.84 *	0.08	−0.80	−0.85 *	−0.81	−0.89 *	−0.34

* indicates significant correlations (*p* ≤ 0.05). N = 3; data are presented as mean ± stand deviation.

**Table 4:** Absorption peak intensity as a function of the wavelength and the pyrolysis temperature of eucalyptus wood biochar.

FT-IR Peak (cm^−1^)	Pyrolysis Temperature (°C)						Vibration	References
	450	550	650	750	850	950		
3.424	Strong	Medium	Weak	Medium	Medium	Weak	O–H Alcohol or phenol	[56]
2.920	Weak	Weak	-	-	-	-	CH_2_–CH_3_ Aliphatic	[52]
1.638	Weak	Weak	Medium	Strong	Strong	Strong	C=C	[52]
1.604	Medium	Medium	Weak	-	-	-	C=O/C=C Aromatics, ketones	[55]
1.430	Strong	Strong	Weak	Weak	Weak	Weak	C–H carboxymethyl-cellulose	[57]
870	Weak	Weak	Strong	Weak	Weak	Weak	C–H in polycyclic aromatic	[58]
